# The prognostic role of desmoplastic stroma in pancreatic ductal adenocarcinoma

**DOI:** 10.18632/oncotarget.6770

**Published:** 2015-12-26

**Authors:** Lai Mun Wang, Michael A. Silva, Zenobia D'Costa, Robin Bockelmann, Zahir Soonawalla, Stanley Liu, Eric O'Neill, Somnath Mukherjee, W. Gillies McKenna, Ruth Muschel, Emmanouil Fokas

**Affiliations:** ^1^ Department of Pathology, Oxford University Hospital NHS Trust, University of Oxford, Oxford, UK; ^2^ Department of Surgery, Oxford University Hospital NHS Trust, Oxford, UK; ^3^ Department of Oncology, CRUK/MRC Institute for Radiation Oncology, University of Oxford, Oxford, UK; ^4^ Department of Radiation Oncology, Sunnybrook Research Institute, Sunnybrook Health Sciences Centre, University of Toronto, Toronto, Canada

**Keywords:** desmoplasia, stroma density, αSMA, pancreatic cancer, prognosis

## Abstract

Pancreatic ductal adenocarcinoma (PDAC) is characterized by an abundant desmoplastic stroma. We examined the prognostic value of stroma density and activity in patients with resectable PDAC treated with surgery and adjuvant gemcitabine-based chemotherapy. FFPE-tissue from the pancreatectomy of 145 patients was immunohistochemically stained for haematoxylin-eosin and Masson's trichrome to assess stroma density, and alpha-smooth muscle actin (αSMA) expression for activated pancreatic stellate cells. Their expression was correlated with clinicopathological characteristics as well as overall survival (OS), progression-free survival (PFS), local progression-free survival (LPFS) and distant metastases free-survival (DMFS). After a mean follow-up of 20 months (range, 2–69 months), the median OS was 21 months and the 3-year OS was 35.7%. In multivariate analysis, highly-dense stroma was an independent prognostic parameter for OS (*p* = 0.001), PFS (*p* = 0.007), LPFS (*p* = 0.001) and DMFS (*p* = 0.002), while αSMA expression lacked significance. Interestingly, highly-dense stroma retained significance for the four clinical endpoints only in early (pT1–2) but not late (pT3–4) stage tumors. Additionally, late pT-stage (pT3–4), the presence of lymph node metastases (pN+ vs pN0), perineural/neural invasion and administration of adjuvant chemotherapy also correlated with prognosis in multivariate analysis. Altogether, stroma density constitutes an independent prognostic marker in PDAC patients treated with adjuvant chemotherapy. Our findings highlight the dynamic complexity of desmoplasia and indicate that highly-dense stroma is correlated with better outcome. Further validation of the prognostic value of stroma as a biomarker and its role in PDAC patients after adjuvant chemotherapy is warranted and will be performed in a prospective study.

## INTRODUCTION

Pancreatic ductal adenocarcinoma (PDAC) has a poor prognosis and very little progress has been made in the past few decades despite the improved understanding of the molecular events underlying the disease [1, 2]. PDAC is characterised by an abundant desmoplastic stroma composed of activated stellate cells (also known as myofibroblasts or cancer-associated-fibroblasts) that express alpha-smooth muscle actin (αSMA) and a large amount of extracellular matrix (ECM) [3, 4]. Traditionally, the desmoplastic stroma has been considered as a dynamic compartment where interactions between cancer and stromal cells promote tumor formation, invasion and metastasis that result in an aggressive disease phenotype [3, 4]. Also, the desmoplastic stroma acts a physical barrier that prevents adequate delivery of chemotherapy impairing its therapeutic potential, and also directly mediates radioresistance [5–7].

Previous histopathological reports have demonstrated an adverse prognostic role for αSMA-positive myofibroblasts in various cancers, such as pancreatic, esophageal, colorectal, breast, head and neck and ovarian cancer [8–13]. The impact of ECM in the clinical setting remains less well investigated [14] despite the ample preclinical evidence supporting a protumorigenic role [15]. Intriguingly, two recent preclinical studies using genetically-engineered mouse models of PDAC showed that depletion of the stroma resulted in even more aggressive and metastatic tumors [8, 16]. These reports contradicted the notion that desmoplasia promotes tumor aggressiveness, indicating that, in certain cases, the stroma could represent a mechanical barrier imposed by the host to limit tumor growth and/or spread [17]. It should be noted, however, that the prognostic impact of stromal density/maturity have been less well explored, which is largely surprising considering the strong interest on the biology of stroma during the last decades. Indeed, in the few studies that examined in detail the prognostic impact of the stroma according to its density, patients with high density presented significantly better outcome compared to patients with intermediate or low stromal density [9, 18, 19]. Interestingly, ECM is mainly produced by activated pancreatic stellate cells but there is often intratumoral variability with lack of linear correlation between the amount of ECM and the degree of myofibroblast activation [13, 19–21]. These contradicting findings highlight the complexity of the stroma and the urgent need to further elucidate its clinical relevance.

Although previous studies have investigated the prognostic impact of the stroma in patients with primarily resectable PDAC, the majority of the reports were characterized by small sample size. In the present study we aimed to evaluate the prognostic significance of stromal density and stromal activity using immumohistochemical staining for haematoxylin-eosin with Masson's trichrome, and αSMA, respectively, in a relatively large cohort (*n* = 145) of patients with PDAC treated with surgery followed by gemcitabine-based chemotherapy.

## RESULTS

### Stroma density and αSMA staining characteristics

**Figure 1:**
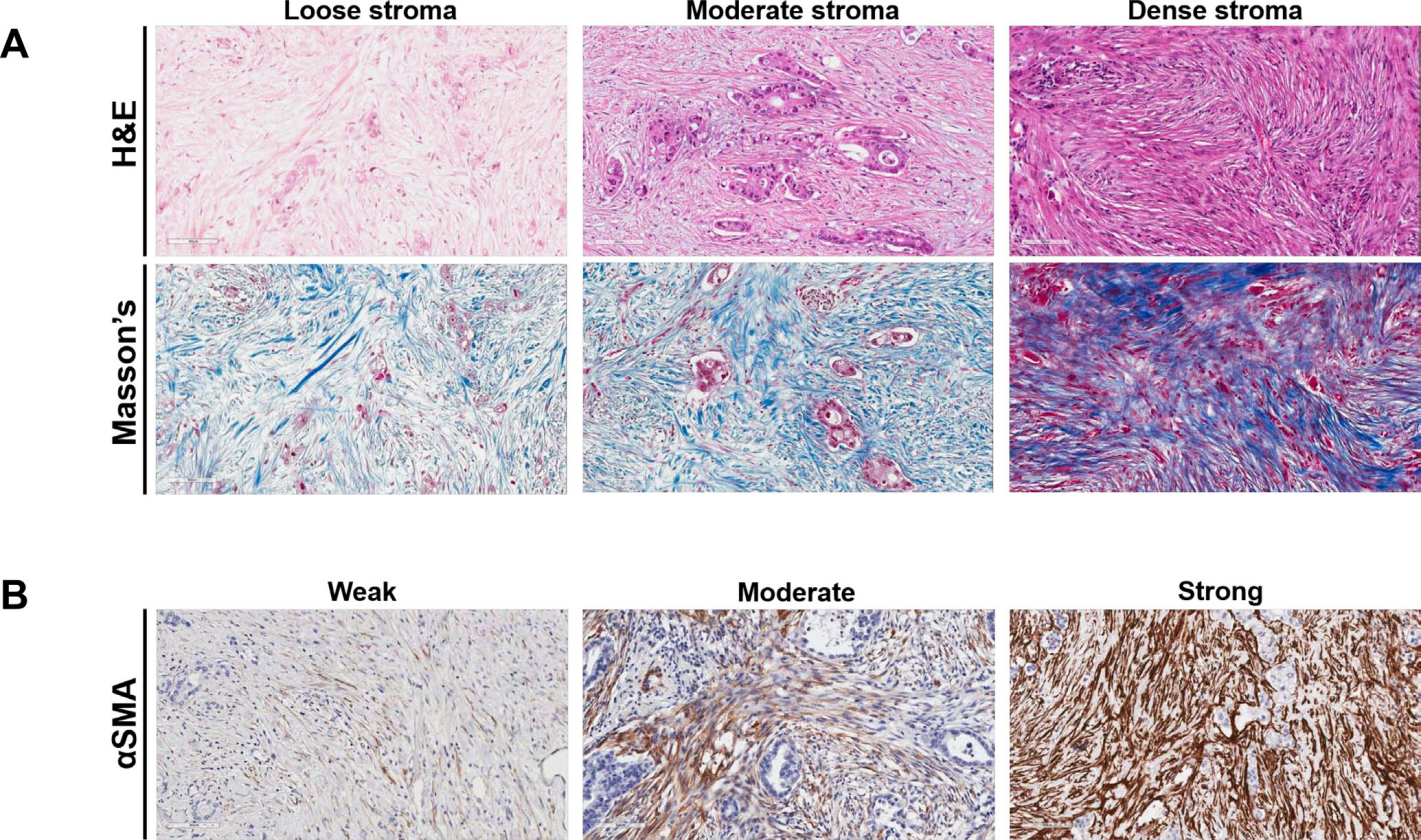
Immunohistochemical staining of desmoplastic stroma in patients with pancreatic ductal adenocarcinoma (**A**) Representative examples of loose, moderate and dense stroma based on H & E staining pattern, as indicated. The corresponding Masson's trichrome staining (blue colour) is shown (same cases as in Figure 1A). (**B**) Representative examples of tumors with weak, moderate and strong expression of alpha smooth muscle actin (αSMA), as indicated. Magnification, x200.

With regard to its density as assessed by H & E, stroma was defined as “loose” in 30 patients (20.7%), “moderate” in 90 patients (62.1%) and “dense” in 25 patients (17.2%), according to the pathologist scoring system. Notably, although Masson's trichrome enabled detection of collagen fibrils (blue colour), the staining pattern did not allow distinction of the different degrees of stroma density as with H & E staining. Indeed, although H & E facilitated clear distinction between loose and moderate stroma density, we could not distinguish loose from moderate-density stroma, based on Masson's trichrome as the amount of collagen was comparable in those 2 stroma groups. Hence, Masson's trichrome was not used in the statistical analysis as we failed to observe a linear correlation between stromal density. Of note, we only found good correlation between H & E and Masson's trichrome staining in the highly-dense stroma group. Hence, stroma density assessment was based entirely on H & E staining patterns. Additionally, αSMA was “negative or weak” in 32 patients (22.1%), “moderate” in 77 patients (53.1%) and “strong” in 36 patients (24.88%). For the purpose of the study analysis, patients with negative or weak expression were classified as “low” (*n* = 32; 22.1%), whereas patients with moderate or strong were defined as “high” (*n* = 113; 77.9%) αSMA expression subgroup. We did not detect a significant association between stroma density and αSMA expression (*p* = 0.370). Dense stroma correlated strongly with G1 tumors (Table 1). Similarly, tumors with low αSMA expression were significantly associated with higher incidence of low-grade (G1) and lower incidence of high-grade (G2–3) tumors. We failed to identify any further significant relationship between either stroma density or αSMA expression and clinicopathologic parameters (Table 1). Of note, we failed to observe tumor cell budding, defined by the presence of small clusters of dispersed cancer cells, in tumours with high density. In contrast, in specimens with either moderate or loose stroma density tumor budding was commonly encountered. Representative examples of loose, moderate and dense stroma as well as weak, moderate and strong αSMA expression are shown in Figure 1. The clinicopathological characteristics for the entire cohort are shown in Supplementary Table 1.

**Table 1:** Clinicopathological characteristics

	αSMA			Stromal Density			
	Negative + Weak *n* (%)	Moderate + Strong *n* (%)	*p*-value	Loose *n* (%)	Moderate *n* (%)	Strong *n* (%)	*p*-value
**Age**							
< median (65 years)	10 (31.3%)	53 (46.9%)	0.084	11 (36.7%)	39 (43.3%)	13 (52%)	0.520
≥ median	22 (68.8%)	60 (53.1%)		19 (63.3%)	51 (56.7%)	12 (48%)	
**Gender**							
Male	19 (59.4%)	49 (43.4%)	0.081	17 (56.7%)	39 (43.3%)	12 (48%)	0.445
Female	13 (40.6%)	64 (56.6%)		13 (43.3%)	51 (56.7%)	13 (52%)	
**Tumor site**							
Head	27 (84.4%)	93 (82.3%)	0.510	25 (83.3%)	74 (82.2%)	21 (84%)	0.974
Other	5 (15.6%)	20 (17.7%)		5 (16.7%)	16 (17.8%)	4 (16%)	
**pT-staging**							
pT1–2	18 (56.3%)	70 (61.9%)	0.350	18 (60%)	52 (57.8%)	18 (72%)	0.435
pT3–4	14 (43.8%)	43 (38.1%)		12 (40%)	38 (42.2%)	7 (28%)	
**pN-staging**							
pN0	10 (31.3%)	25 (22.1%)	0.201	6 (20%)	19 (21.1%)	10 (40%)	0.125
pN+	22 (68.8%)	88 (77.9%)		24 (80%)	71 (78.9%)	15 (60)	
**Grading**							
G1	7 (21.9%)	1 (0.9%)	**< 0.001**	1 (3.3%)	4 (4.4%)	3 (12%)	**0.044**
G2	19 (59.4%)	75 (66.4%)		14 (46.7%)	63 (70%)	17 (68%)	
G3	6 (18.8%)	37 (32.7%)		15 (50%)	23 (25.6%)	5 (20%)	
**Resection margins**							
R0	15 (46.9%)	39 (34.5%)	0.143	8 (26.7%)	34 (37.8%)	12 (48%)	0.261
R1	17 (53.1%)	74 (65.5%)		22 (73.3%)	56 (62.2%)	12 (52%)	
**Type of surgery**							
Whipples	16 (50%)	76 (67.3%)	0.190	18 (60%)	58 (64.4%)	16 (64%)	0.959
Pylorus preserving	11 (34.4%)	27 (23.9%)		8 (26.7%)	24 (26.7%)	6 (24%)	
Total pancreatectomy	5 (15.6%)	10 (8.8%)		4 (13.3%)	8 (8.9%)	3 (12%)	
**PNI**							
no	28 (87.5%)	86 (76.1%)	0.224	24 (80%)	71 (78.9%)	19 (76%)	0.932
yes	4 (12.5%)	27 (23.9%)		6 (20%)	19 (1.1.9%)	6 (24%)	
**VI**							
no	15 (46.9%)	37 (32.7%)	0.150	11 (36.7%)	30 (33.3%)	11 (44%)	0.613
yes	17 (53.1%)	76 (67.3%)		19 (63.3%)	60 (66.7%)	14 (56%)	
**LI**							
no	10 (31.3%)	43 (38.1%)	0.313	11 (36.7%)	28 (31.1%)	14 (56%)	0.073
yes	22 (68.8%)	70 (61.9%)		29 (63.3%)	62 (68.9%)	11 (44%)	
**Chemotherapy**							
No	2 (6.3%)	17 (15%)	0.107	4 (13.3%)	9 (10%)	6 (24%)	0.102
1–2 cycles	4 (12.5%)	28 (24.8%)		8 (26.7%)	23 (25.6%)	1 (4%)	
≥ 3 cycles	26 (81.3%)	68 (60.2%)		18 (60%)	58 (64.4%)	18 (72%)	

Abbreviations: VI, vascular invasion; LI, lymphatic invasion; PNI, perineural/neural invasion; significant results have been marked with bold.

### Stroma immunostaining and clinical outcome

**Figure 2:**
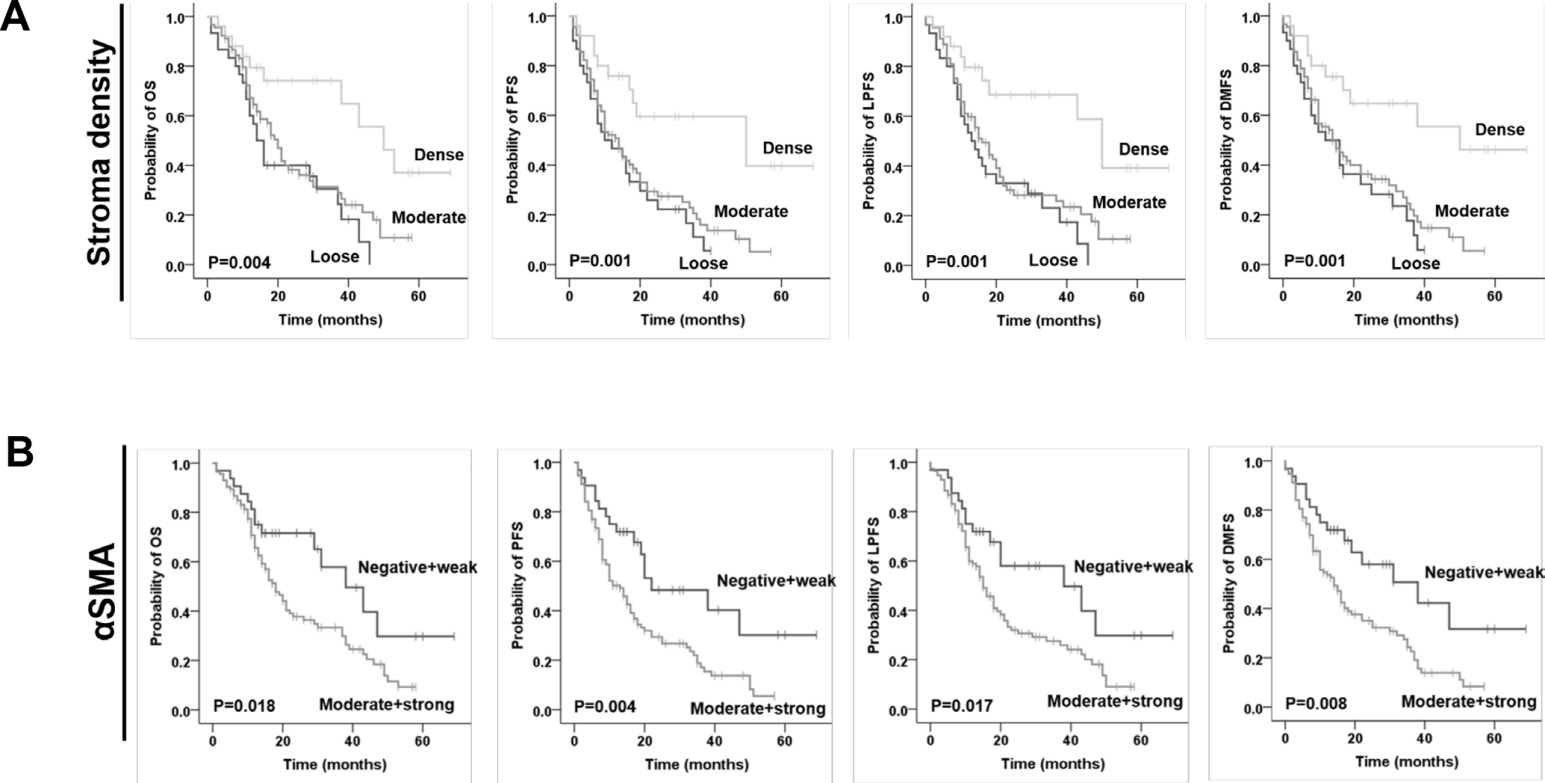
Prognostic impact of (**A**) stroma density based on H & E staining and (**B**) alpha smooth muscle actin (αSMA) on overall survival (OS), progression-free survival (PFS), local progression-free survival (LPFS) and distant metastases free survival (DMFS) in patients with pancreatic ductal adenocarcinoma, as indicated.

After a mean follow-up of 20 months (range, 2–69 months), the median OS was 21 months and the 3-year OS was 35.7% for the entire patient cohort. From the 145 patients, 15 (10.35%) developed local recurrence, 56 (38.6%) developed distant recurrence, 15 (10.35%) had both local and distant recurrence, whereas 59 (40.7%) had no recurrence by the time of analysis. Patients with dense stroma had a significantly superior OS (dense vs moderate vs loose stroma: mean 45.0 vs 25.2 vs 21.4 months; *p* = 0.004), PFS (dense vs moderate vs loose stroma: mean 41.8 vs 19.3 vs 15.6 months; *p* = 0.001), LPFS (dense vs moderate vs loose stroma: mean 44.4 vs 22.8 vs 19.4 months; *p* = 0.001) and DMFS (dense vs moderate vs loose stroma: mean 43.6 vs 20.9 vs 17.0 months; *p* = 0.001) in univariate analysis (Figure 2A and Table 2). Additionally, patients with moderate/strong αSMA expression were characterized by a significantly worse OS (absent/weak vs moderate/strong αSMA: mean 39.2 vs 24.7 months; *p* = 0.018), PFS (absent/weak vs moderate/strong αSMA: mean 37.4 vs 22.6 months; *p* = 0.017) and DMFS (absent/weak vs moderate/strong αSMA: mean 36.7 vs 20.5 months; *p* = 0.008) (Figure 2B and Table 2). Tumor grading significantly affected PFS (*p* = 0.003), LPFS (*p* = 0.022) and DMFS (*p* = 0.002) and presented a trend towards significance for OS (*p* = 0.060). Univariate analyses also revealed a significant impact for pT-stage, pN-stage, resection margins, perineural/neural invasion (PNI) and venous invasion (VI) for all clinical endpoints (Table 2). We also confirmed the prognostic impact of stroma density by comparing the different density groups between them (loose vs moderate; loose vs highly-dense; moderate vs highly-dense) for all four clinical endpoints (Supplementary Table 2).

**Table 2:** Univariate and multivariate analysis of prognostic factors

	Univariate		Multivariate		
	*p*-value	HR	95% CI		*p*-value
			Lower	Upper	
**OS**					
Stroma density (loose vs moderate vs dense)	**0.004**	0.522	0.355	0.767	**0.001**
aSMA (negative + weak vs moderate + strong)	**0.018**	1.216	0.630	2.349	0.560
Age (< median(65) vs ≥ median)	0.556	1.072	0.657	1.748	0.781
Sex (male vs female)	0.604	1.508	0.907	2.506	0.113
Tumour localisation (head vs other)		0.732	0.352	1.525	0.405
pT-stage (pT1–2 vs pT3–4)	**0.001**	1.520	1.032	2.238	**0.034**
pN-stage (pN0 vs pN+)	**0.001**	1.526	0.781	2.982	0.216
Grading (G1 vs G2 vs G3)	0.060	0.935	0.577	1.517	0.786
Resection margins (R0 vs R1)	**0.001**	1.167	0.661	2.058	0.595
Type of surgery (W vs PP vs TP)	0.848	0.976	0.646	1.475	0.908
PNI (no vs yes)	**0.001**	2.084	1.269	3.421	**0.004**
VI (no vs yes)	**0.006**	1.467	0.813	2.646	0.203
LI (no vs yes)	0.112	0.782	0.450	1.358	0.383
Chemotherapy (no vs 1–2 cycles vs ≥ 3 cycles)	**< 0.001**	0.445	0.321	0.618	**0.001**
**PFS**					
Stroma density (loose vs moderate vs dense)	**0.001**	0.613	0.430	0.874	**0.007**
aSMA (negative + weak vs moderate + strong)	**0.004**	1.282	0.705	2.331	0.415
Age (< median(65) vs ≥ median)	0.617	1.090	0.690	1.720	0.713
Sex (male vs female)	0.753	1.254	0.788	1.997	0.339
Tumour localisation (head vs other)	0.311	0.842	0.440	1.613	0.605
pT-stage (pT1–2 vs pT3–4)	**0.001**	1.450	1.011	2.078	**0.043**
pN-stage (pN0 vs pN+)	**0.001**	2.120	1.121	4.010	**0.021**
Grading (G1 vs G2 vs G3)	**0.003**	1.177	0.757	1.831	0.469
Resection margins (R0 vs R1)	**0.001**	1.208	0.713	2.046	0.482
Type of surgery (W vs PP vs TP)	0.430	0.873	0.602	1.267	0.475
PNI (no vs yes)	**< 0.001**	2.292	1.422	3.695	**0.001**
VI (no vs yes)	**0.005**	1.290	0.762	2.186	0.343
LI (no vs yes)	0.060	0.814	0.496	1.337	0.417
Chemotherapy (no vs 1–2 cycles vs ≥ 3 cycles)	**< 0.001**	0.588	0.431	0.801	**0.001**
**LPFS**					
Stroma density (loose vs moderate vs dense)	**0.001**	0.539	0.370	0.785	**0.001**
aSMA (negative + weak vs moderate + strong)	**0.017**	1.231	0.654	2.319	0.520
Age (< median(65) vs ≥ median)	0.265	1.347	0.834	2.174	0.223
Sex (male vs female)	0.437	1.474	0.914	2.376	0.112
Tumour localisation (head vs other)	0.221	0.815	0.399	1.665	0.574
pT-stage (pT1–2 vs pT3–4)	**0.005**	1.408	0.971	2.041	0.071
pN-stage (pN0 vs pN+)	**0.001**	1.609	0.833	3.108	0.157
Grading (G1 vs G2 vs G3)	**0.022**	0.941	0.593	1.494	0.796
Resection margins (R0 vs R1)	**0.001**	1.319	0.763	2.282	0.321
Type of surgery (W vs PP vs TP)	0.545	0.910	0.607	1.366	0.650
PNI (no vs yes)	**< 0.001**	2.507	1.526	4.117	**0.001**
VI (no vs yes)	**0.005**	1.371	0.770	2.440	0.284
LI (no vs yes)	0.083	0.817	0.483	1.384	0.453
Chemotherapy (no vs 1–2 cycles vs ≥ 3 cycles)	**< 0.001**	0.510	0.372	0.700	**0.001**
**DMFS**					
Stroma density (loose vs moderate vs dense)	**0.001**	0.561	0.388	0.811	**0.002**
aSMA (negative + weak vs moderate + strong)	**0.008**	1.266	0.684	2.345	0.453
Age (< median(65) vs ≥ median)	0.914	0.979	0.613	1.564	0.930
Sex (male vs female)	0.438	1.421	0.879	2.296	0.151
Tumour localisation (head vs other)	0.316	0.841	0.430	1.644	0.612
pT-stage (pT1–2 vs pT3–4)	**0.001**	1.385	0.958	2.004	0.084
pN-stage (pN0 vs pN+)	**0.001**	2.064	1.080	3.943	**0.028**
Grading (G1 vs G2 vs G3)	**0.002**	1.123	0.713	1.768	0.617
Resection margins (R0 vs R1)	**0.001**	1.161	0.672	2.006	0.593
Type of surgery (W vs PP vs TP)	0.387	0.839	0.567	1.243	0.382
PNI (no vs yes)	**0.001**	2.029	1.254	3.283	**0.004**
VI (no vs yes)	**0.004**	1.413	0.827	2.414	0.206
LI (no vs yes)	0.076	0.801	0.480	1.336	0.395
Chemotherapy (no vs 1–2 cycles vs ≥ 3 cycles)	**< 0.001**	0.551	0.399	0.761	**0.001**

Abbreviations: HR, hazard ratio; CI, confidence interval; W, Whipples; PP, partial pancreatectomy; TP, total pancreatectomy; VI, vascular invasion; LI, lymphatic invasion; PNI, perineural/neural invasion; OS, overall survival; PFS, progression-free survival; FFS, local failure-free survival; DMFS, distant metastases-free survival.

Significant values have been marked with bold.

We performed a multivariate analysis by including the two stroma markers and the clinicopathological factors (Table 2). In the Cox model, highly-dense stroma was confirmed as an independent prognostic parameter for OS (*p* = 0.001), PFS (*p* = 0.007), LPFS (*p* = 0.001) and DMFS (*p* = 0.002), whereas no significance was found for αSMA expression. Similarly, late pT-stage (pT3–4 vs pT1–2) was associated with worse OS (*p* = 0.034) and PFS (*p* = 0.043), whereas the presence of lymph node metastases (pN^+^ vs pN0) correlated with worse PFS (*p* = 0.021) and DMFS (*p* = 0.028). Interestingly, PNI and administration of adjuvant chemotherapy retained their significance for all four clinical endpoints in the multivariate analysis (Table 2).

**Figure 3:**
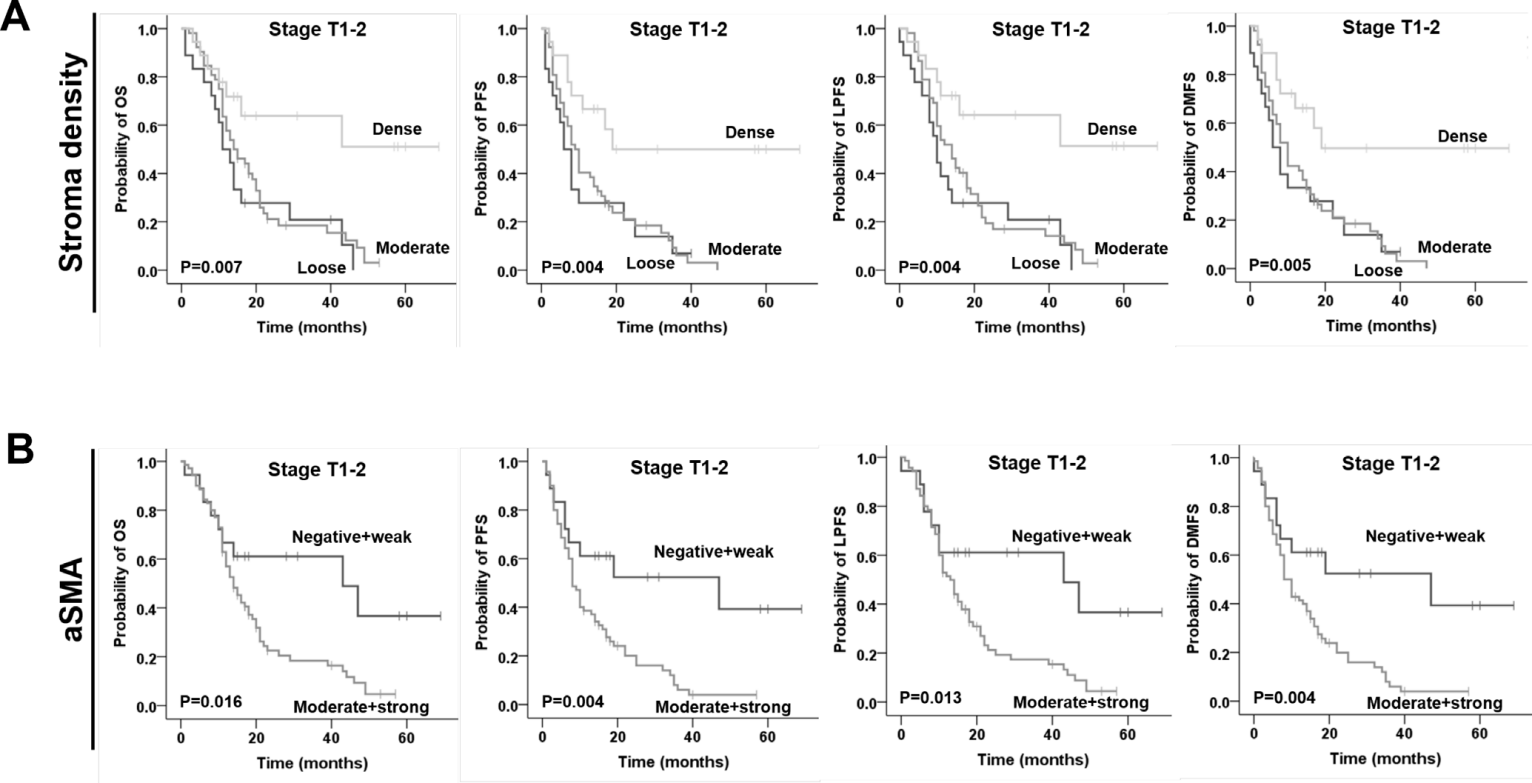
Prognostic impact of (**A**) stroma density based on H & E staining and (**B**) alpha smooth muscle actin (αSMA) on overall survival (OS), progression-free survival (PFS), local progression-free survival (LPFS) and distant metastases free survival (DMFS) in patients with early stage (pT1–2) pancreatic ductal adenocarcinoma, as indicated. Only significant results are shown.

Furthermore, we examined the prognostic significance of stroma density and αSMA according to the tumor size (pT1–2 vs pT3–4; Figure 3; Table 3). Intriguingly, patients with dense stroma had a significantly better clinical outcome only in early (pT1–2) but not late (pT3–4) stage tumors (OS: *p* = 0.007; PFS: *p* = 0.004; LPFS: *p* = 0.004; DMFS: *p* = 0.005). Similarly, moderate and strong αSMA expression was associated with a less favourable outcome only in patients with stage pT1–2 tumors (OS: *p* = 0.016; PFS: *p* = 0.004; LPFS: *p* = 0.013; DMFS: *p* = 0.004), whereas no significance was detected for pT3–4 disease (Figure 3, Table 3).

**Table 3:** Prognostic impact of stroma based on pT-stage

Stroma marker and T-stage	OS *p*-value	PFS *p*-value	LPFS *p*-value	DMFS *p*-value
**Stroma density**				
Stage pT1–2	**0.007**	**0.004**	**0.004**	**0.005**
Stage pT3–4	0.123	0.153	0.199	0.067
**αSMA**				
Stage pT1–2	**0.016**	**0.004**	**0.013**	**0.004**
Stage pT3–4	0.961	0.590	0.659	0.899

Abbreviations: OS, overall survival; PFS, progression-free survival; LFFS, local failure-free survival; DMFS, distant metastases-free survival; significant values have been marked with bold.

## DISCUSSION

Although previous studies have assessed the impact of desmoplasia on the clinical outcome in patients with PDAC, the prognostic value of stroma density in patients with PDAC remains less well explored. In the present work, patients with high stromal density had a significantly better clinical outcome compared to patients with intermediate or low stromal density in multivariate analysis. This finding was independent of clinicopathological parameters with a predictive role in this tumor type. Of note, pT stage, pN stage, PNI and chemotherapy also presented statistical significance for the clinical outcome in our series, although variability was observed.

We failed to detect prognostic significance for αSMA in multivariate analysis in our cohort. αSMA is mainly produced by activated pancreatic stellate cells (PSCs) that resemble myofibroblasts and are the main source of stroma production in PDAC [24]. Co-culture and animal experiments have previously demonstrated that activated αSMA-positive PSCs decrease response to chemotherapy and radiotherapy and promote tumor invasion and metastases [6, 25–29]. Previous studies have examined the prognostic value of αSMA expression for the clinical outcome of patients with various tumor types. In PDAC, Fujita et al. showed that high αSMA mRNA levels correlated with worse outcome in 109 patients that received surgery but this cohort was characterised by high heterogeneity as five different adjuvant chemotherapy regimens were administered [30]. Similar findings were reported in the analysis of the CONKO-001 cohort of 162 patients [9]. Additionally, Herrera et al. and Marsh et al. demonstrated an adverse prognostic role for strong αSMA expression in 289 and 282 patients with colorectal and oral cancer, respectively [10, 12]. In contrast to the above findings, a recent work by Kalluri and colleagues revealed improved outcome for high αSMA expression in 53 patients with PDAC [8]. Genetic depletion of αSMA-positive myofibroblasts in a genetically-engineered mouse model (GEMM) of PDAC was associated with decreased ECM content and led to increased tumor progression and decreased survival [8]. Stanger and colleagues reported similar findings after genetic or pharmacologic depletion of sonic hedgehog in a different GEMM that led to reduced desmoplasia associated with accelerated tumor growth and metastasis [16]. In contrast, the Hopkins group failed to identify a prognostic significance for αSMA in 66 patients that received adjuvant chemotherapy with or without vaccine therapy [20]. Erkan et al. showed that the combination of high αSMA and low collagen expression, defined as activated stroma index, was associated with worse outcome but αSMA alone lacked a prognostic role [13]. There are several potential reasons for the incoherent data regarding αSMA and outcome. First, tthe different findings could be attributed to the inhomogeneous treatment regimens and patient cohorts. Second, the small patient number (*n* = 53) used by Kalluri and colleagues could be associated with bias due to the lower statistical power [8]. Third, although widely studied, the KRAS^LSL.G12D/+^; p53^R172H/+^; PdxCre^tg/+^ (KPC) GEMM used in their study only represents a proportion of patients as p53 mutations are found in approximately 50% of patients with PDAC [31]. Fourth, several previous studies examined αSMA in small sections, including tissue microarrays (diameter 1–2 mm), instead of the entire pancreatectomy section, as in our work. This is crucial point as small sections do not allow assessment of tumour heterogeneity and could lead to potential bias in histological scoring. Hence, prospective studies based on well-defined pathology criteria in entire pancreatectomy sections are required to better elucidate the prognostic significance of αSMA in PDAC.

Patients with tumors of high stroma density had significantly improved outcome in the present analysis. Of note, H & E staining enabled us to better assess stroma density as compared with Masson's trichrome that only detects collagen fibrils. To our knowledge, only the CONKO-001 group has previously assessed stroma density using the three-tier classification (loose vs moderate vs dense stroma) in PDAC [9]. In that work, high stroma density correlated with better outcome compared to moderate or loose density stroma. The Hopkins group reported longer survival in patients with high stroma density but the latter was assessed using a dichotomized system based on collagen staining only in a small cohort that was likely to be underpowered (*n* = 59) [20]. In two elegant studies, Ueno et al. also used H & E staining and observed superior survival rates in rectal cancer and liver metastases patients with highly-dense stroma [18, 19]. Hence, our findings confirmed the CONKO-001 report in PDAC and are in line to previous observations in other malignancies.

The classical paradigm of tumor micromilieu has supported the notion that stroma promotes tumor growth and progression [32]. On the other hand, it has been hypothesised that the desmoplastic stroma constitutes a reactive defence mechanism of the host to confine inflammation, such as pancreatitis, prevent progression of premalignant lesions to invasive carcinoma and/or mechanically prevent tumor cells from spreading to distant organs, similarly to wound healing [33–36]. Our findings support this hypothesis as high stroma density was associated with a significantly longer survival in small (pT1–2) but not large tumors that have spread beyond the pancreas (pT3–4) and was closely correlated with higher incidence of low-grade (G1) tumors in our cohort. This is an important finding as several studies, including a Surveillance, Epidemiology, and End Results database in 8082 patients with resected PDAC showed that G1 is a independent prognostic parameter for better outcome [37, 38], highlighting the correlation of high stromal density with a more favourable diseasephenotype This is, to our knowledge, the first work to investigate the prognostic value of stromal density in small (pT1–2) vs large (pT3–4) with the outcome. An older work found that tumors cells with strong ability to induce a stromal response had a lower ability to metastasize and vice versa [39]. Furthermore, pathology series have demonstrated the presence of only moderate and loose density stroma at the invasive front in colorectal cancer, whereas mature dense stroma was mainly confined in tumor centre [18, 40]. The potential contradiction between better outcome of dense stroma-containing tumors and worse outcome of highly αSMA-positive tumors could explained by the lack of stroma uniformity. Also, αSMA-positive PSCs activation can occur in chronic pancreatitis-like changes with ECM deposition that can confine the inflammatory process [21]. Intriguingly, Kadaba et al. mixed different proportions of immortalized PSCs that stain positive for a SMA and PDAC cells (10:1–1:10) and showed that the maximum cancer cell proliferation and invasion occurred when PSCs represented the majority of the cell population in the 3D model, highlighting the complexity of the stroma [28]. Interestingly, tumors with unfavourable less dense fibrotic stroma patterns were characterised by extensive tumor cell budding, which is in line to our observations. Of note, tumor buds show strong expression of the epithelial-mesenchymal transition markers ZEB1 and ZEB2 [41]. Thus, it is likely that loose and moderate density stroma allows or even facilitates dedifferentiation of cancer cells, promoting tumor progression and metastasis.

Our data raise caution regarding the implementation of stroma-depleting agents as an emerging paradigm in the treatment of PDAC since there appears to be lack of linear correlation between the amount of stroma and the clinical outcome. There is currently little evidence to either support or discourage the application of these agents, despite the initially disappointing results using sonic hedgehog inhibitors in PDAC clinical trials [42]. Hence, therapeutic strategies that modify the stroma need careful reconsideration as they might be beneficial only in a certain subgroup of patients with PDAC [43].

Although prospectively treated and followed-up, the retrospective evaluation of the prognostic impact of stroma represents limitation of our study and hence we cannot exclude potential selection bias. This should be taken into consideration when interpreting our findings and hence confirmation in more prospective studies is warranted. Additionally, the median follow-up in the present study is relatively short compared to previous reports. Furthermore, the stromal density and αSMA were calculated using a non-automated method due to the lack of standardized scoring system. The use of a manual scoring system results in the samples being binned, and the prognostic significance determined by crude positive and negative categories, whereas the intensities of stroma and aSMA staining are probably continuous variables. Nevertheless, our observations highlight the complexity of the stroma and emphasize the importance of detailed analysis of stromal density since quantitative assessment of stroma markers by using dichotomization methods might provide insufficient information.

In summary, stromal density represents a promising prognostic marker to identify PDAC patients with a more favourable clinical outcome after adjuvant chemotherapy. The current findings highlight the complex nature of the desmoplastic stroma in PDAC and could have direct translational implications for future clinical studies with standard treatment and stromal-modifying agents. Our findings warrant validation in prospective cohorts before routine use of stromal density as a biomarker in the clinical setting.

## METHODS

### Patients and treatment

Patients were treated between 2009 and 2014 with surgery and postoperative chemotherapy at the Oxford University Hospital NHS Trust, Oxford, UK. The type of pancreatectomy conducted was according to well-established international criteria. Patients included in this retrospective study were required to meet the following criteria: previous complete macroscopic surgical resection (R0 or R1) for histologically-confirmed PDAC, absence of metastatic spread to the liver or other intra- or extraabdominal organs, absence of malignant ascites, absence of secondary tumors, no previous treatment, availability of formalin-fixed paraffin-embedded (FFPE) tissue blocks in the pathology archive stored under conditions that enabled tissue preservation.

Regarding chemotherapy, the majority of patients received gemcitabine alone (GEM), whereas a small proportion received chemotherapy with a combination of gemcitabine with capecitabine (GEM-CAP). GEM alone was administered via intravenous infusion at a dose of 1,000 mg/m^2^ over 30 minutes, at days 1, 8 and 15 (1 cycle) for in total 6 cycles. Patients treated with the GEM-CAP regimen received GEM as above, whereas CAP was given orally at a dose 1,660 mg/m^2^/d (830 mg/m^2^ twice daily) for 3 weeks followed by 1 week pause. In total, *n* = 145 patients that fulfilled all criteria were included in the study. Archived FFPE tissue specimens were acquired together with clinical follow-up data and diagnostic images at the Oxford University Hospital NHS Trust. Informed consent had been obtained and the study was approved by the local ethics committee of the University of Oxford (Project: OCHRe 14/A176).

### Immunohistochemical staining

The best representative FFPE tumour block was selected after reviewing the slides from *n* = 145 pancreatomy specimens available for the study of stromal morphology and 3-μm thick sections were cut and mounted on coated superfrost slides. The best block was chosen based on the best representation of adequate tumour volume i.e. not a block with few tumour cells and lots of background pancreas, including satisfactory processing to ensure minimal artefact and avoid potential interference with immune- and special stains. These were stained with haematoxylin and eosin (H & E) as previously reported [22]. For the immunohistochemical staining of αSMA the Leica Bond Max staining platform was used at the Department of Pathology, Oxford University Hospital NHS Trust. A horseradish-peroxidase technique and a DAKO Autostainer Link 48 (DAKO, UK) were used. Antigen retrieval was accomplished by the pretreatment of the paraffin sections (SuperFrost Plus, Thermo Scientific, UK) with the Bond TM Epitope Retrieval 1-1L Reagent (Leica Microsystems, UK) for 20 min on the Bond Max staining machine. Following that, slides were stained with a primary antibody for αSMA (clone 1A4, dilution 1:1000 in Bond antibody diluent; Dako, UK) following incubation for 180 minutes at room temperature. After that, dextran polymer-conjugated horseradish-peroxidase and 3,3′-diaminobenzidine (DAB) chromogen intensified with 1% copper sulphate was applied followed by a light haematoxylin counterstain (Gill 3, Sigma, UK). Collagen staining was accomplished using the HT-15 Masson's trichrome staining kit (Sigma Aldrich, UK) according to manufacturer's instructions with slight modifications. In brief, slides were pretreated with the Bouin's solution at room temperature overnight followed by nucleus staining with Meyer's Haematoxylin for 8 min. Subsequently, slides were placed in phosphotungstic-phosphomolybdic acid solution for 10 min and finally in aniline blue solution for 10 min. Sections without primary antibodies served as a negative control.

Stromal density based on H & E staining, and classified as loose, moderate or strong as previously described [9, 18, 19]. Loose density stroma is characterised by a loose fibroblastic myxoid stroma and occasional short wispy collagen fibres. Moderate density stroma is composed of interrupted bands of keloid-like collagen (collagen with brightly eosinophilic hyalinisation, similarly to keloid) without myxoid changes. Strong (highly-dense) stroma presents mature collagen fibres i.e. fine elongated collagen fibres densely packed into multilayers with intense staining lacking keloid-like collagen bands. We also examined Masson's trichrome staining pattern in conjunction with H & E staining. Stromal myofibroblastic activity assessment was based on αSMA immunohistochemistry and categorised as negative, weak, moderate or strong. Subsequently, negative and weak expression were combined and regarded as “low αSMA”; whereas moderate and strong were combined as “high αSMA” [9]. Representative images from patients with different stromal densities and αSMA expression are shown in Figure 1.

Images were scanned and analysed at a x20 magnification using the ImageScope Viewer (Aperio Technologies, Inc., Vista, CA, USA). In contrast to the majority of previous studies that have examined stromal morphology in either tissue microarrays or small sections, we performed our analysis using large sections from the entire tumor area that allowed a better assessment of the tissue sample. Thus, our analysis enabled detailed and representative evaluation of the stroma morphology in the entire section. At least ten different areas of the tumor were assessed. Immunohistochemical scoring was established by a board certified pathologist (LMW) with large experience and expertise in gastrointestinal malignancies. Blinded samples were evaluated by two observers (LMW and EF). In case of discrepancy, a final decision was made after further microscopy examination of the slides based on consensus by the two investigators. In cases of intratumoral heterogeneity, the dominant staining pattern was identified and used for scoring.

### Statistics

The Fisher's exact test was used to assess differences between categorical variables. Overall survival (OS) was calculated from the date of surgery to the day of death from any cause. Progression-free survival (PFS) was defined from the date of surgery to the day of local or distant recurrence or death from any cause. Distant metastasis free survival (DMFS) and local progression free survival (LPFS) were calculated from the date of surgery to distant metastasis or death and local progress or death, respectively. Patients that did not develop either local or distant tumor recurrence were censored at the last follow-up contact. Factors with a *p* < 0.05 was considered statistically significant. Survival curves were plotted based on the Kaplan–Meier method. Univariate analyses were conducted using the log-rank (Mantel–Cox) test and multivariate analyses by means of the Cox proportional hazard model. The statistical power of the study was 0.831, which constitutes a “high power” analysis [23]. All statistical analyses were performed using the SPSS 20 software (SPSS Inc., Chicago, IL, USA)

## SUPPLEMENTARY MATERIALS FIGURES AND TABLES


