# Supplementary material for: The prognostic role of desmoplastic stroma in pancreatic ductal adenocarcinoma
Source: Oncotarget. 2015 Dec 26;7(4):4183–94. doi: 10.18632/oncotarget.6770 (PMC4826198; doi:10.18632/oncotarget.6770)
Supplement: Supplementary file 1 [file oncotarget-07-4183-s001.pdf]

The prognostic role of desmoplastic stroma in pancreatic ductal adenocarcinoma

Supplementary Materials

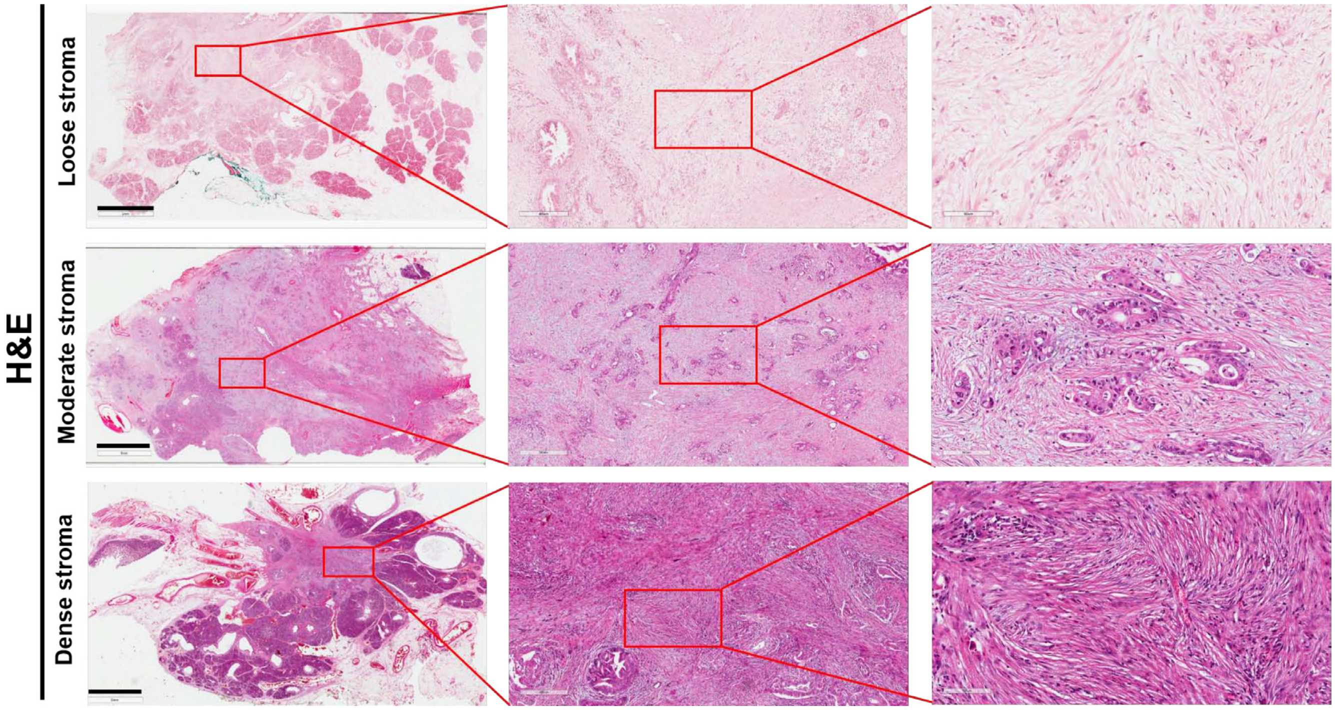

Supplementary Figure S1: Immunohistochemical patterns of desmoplastic stroma density (same sections as in Figure 1A). Stroma was defined as loose, moderate or dense based on H & E staining, as indicated. The left panels illustrate large pancreatectomy sections (Bar: 5 mm). The magnifications of the middle and right inserts are x50 and x200, respectively.

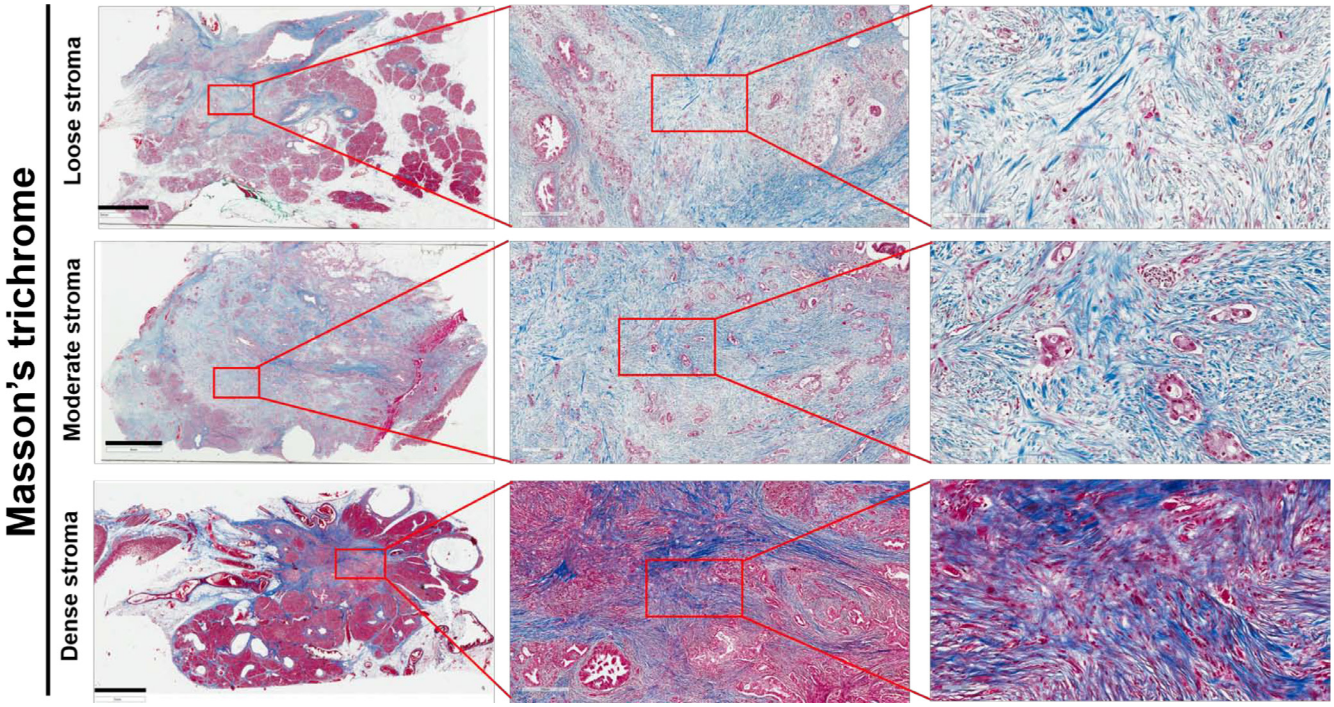

Supplementary Figure S2: Immunohistochemical patterns of desmoplastic stroma collagen (same sections as in Figure 1A). Collagen was stained using Masson's trichrome (blue colour). The left panels illustrate large pancreatectomy sections (Bar: 6 mm). The magnifications of the middle and right inserts are x50 and x200, respectively.

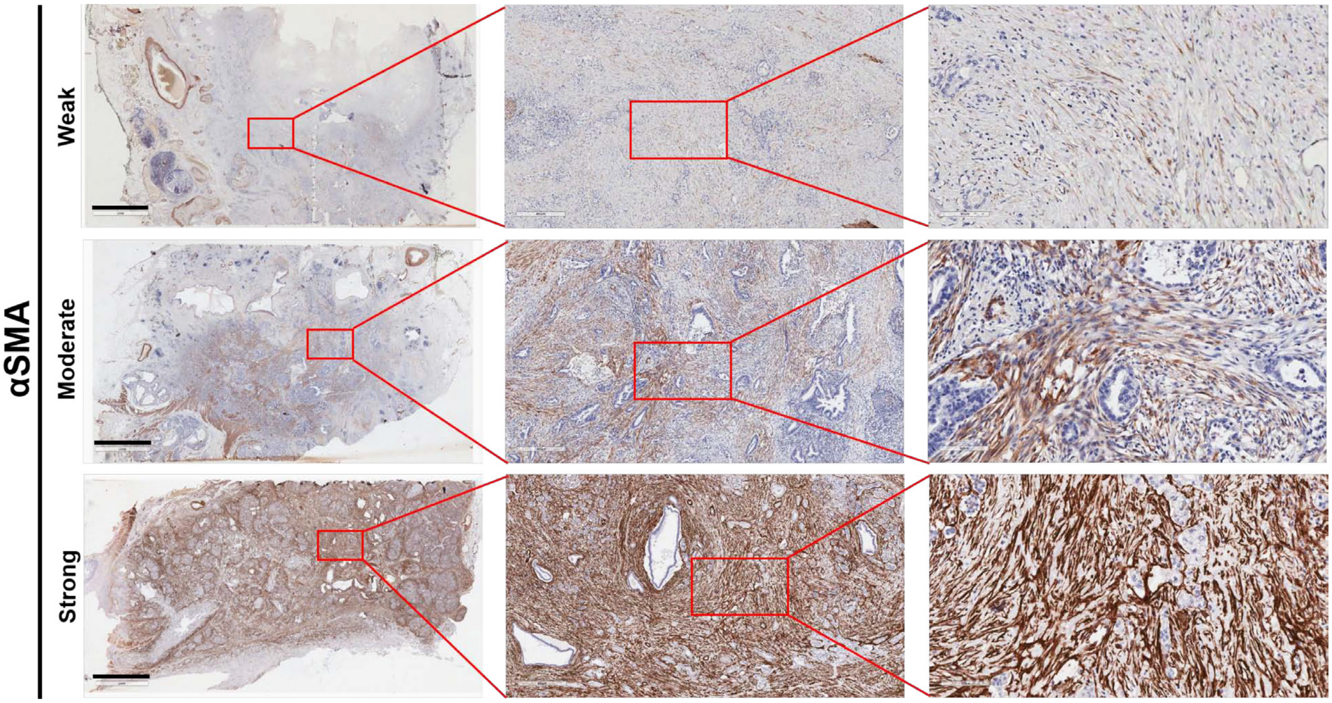

Supplementary Figure S3: Immunohistochemical staining of alpha smooth muscle actin (αSMA) (same sections as in Figure 1B). αSMA was defined as weak/negative, moderate and strong. The left panels illustrate large pancreatectomy sections (Bar: 6 mm). The magnifications of the middle and right inserts are x50 and x200, respectively.

Supplementary Table S1: Clinicopathological characteristics of the cohort

	n (%)
**Age**	
< median (65 years)	63 (43.4%)
≥ median	82 (56.6%)
**Gender**	
Male	68 (46.9%)
Female	77 (53.1%)
**Tumor site**	
Head	120 (82.8%)
Other	25 (17.2%)
**pT-staging**	
pT1–2	88 (60.7%)
pT3–4	57 (39.3%)
**pN-staging**	
pN0	35 (24.1%)
pN+	110 (75.9%)
**Grading**	
G1	8 (5.5%)
G2	94 (64.8%)
G3	43 (29.7%)
**Resection margins**	
R0	54 (37.2%)
R1	91 (62.8%)
**Type of surgery**	
Whipples	92 (63.4%)
Pylorus preserving	38 (26.2%)
Total pancreatectomy	15 (10.4%)
**PNI**	
no	114 (78.6%)
yes	31 (21.4%)
**VI**	
no	52 (35.9%)
yes	93 (64.1%)
**LI**	
no	53 (36.6%)
yes	92 (63.4%)
**Chemotherapy**	
No	19 (13.1%)
1–2 cycles	32 (22.1%)
≥ 3 cycles	94 (64.8%)

Abbreviations: VI, vascular invasion; LI, lymphatic invasion; PNI, perineural/neural invasion;

Supplementary Table S2: Prognostic impact of stroma density

Stroma marker	OS<br>*p*-value	PFS<br>*p*-value	LPFS<br>*p*-value	DMFS<br>*p*-value
**Stroma density**				
Loose vs moderate	0.277	0.396	0.388	0.303
Loose vs highly dense	**0.001**	**0.001**	**0.001**	**< 0.001**
Moderate vs highly dense	**0.007**	**0.001**	**0.002**	**0.003**

Abbreviations: OS, overall survival; PFS, progression-free survival; LFFS, local failure-free survival; DMFS, distant metastases-free survival; significant values have been marked with bold.
